# Uncovering neuroinflammation-related modules and potential repurposing drugs for Alzheimer's disease through multi-omics data integrative analysis

**DOI:** 10.3389/fnagi.2023.1161405

**Published:** 2023-06-02

**Authors:** Shensuo Li, Changhao Lu, Zhenzhen Zhao, Dong Lu, Guangyong Zheng

**Affiliations:** ^1^Shanghai Frontiers Science Center for Chinese Medicine Chemical Biology, Institute of Interdisciplinary Integrative Medicine Research, Shanghai University of Traditional Chinese Medicine, Shanghai, China; ^2^Department of Biomedical Sciences, University of Sassari, Sassari, Italy

**Keywords:** Alzheimer's disease, neuroinflammation, amyloid-β, data integration, drug repurposing

## Abstract

**Background:**

Neuroinflammation is one of the key factors leading to neuron death and synapse dysfunction in Alzheimer's disease (AD). Amyloid-β (Aβ) is thought to have an association with microglia activation and trigger neuroinflammation in AD. However, inflammation response in brain disorders is heterogenous, and thus, it is necessary to unveil the specific gene module of neuroinflammation caused by Aβ in AD, which might provide novel biomarkers for AD diagnosis and help understand the mechanism of the disease.

**Methods:**

Transcriptomic datasets of brain region tissues from AD patients and the corresponding normal tissues were first used to identify gene modules through the weighted gene co-expression network analysis (WGCNA) method. Then, key modules highly associated with Aβ accumulation and neuroinflammatory response were pinpointed by combining module expression score and functional information. Meanwhile, the relationship of the Aβ-associated module to the neuron and microglia was explored based on snRNA-seq data. Afterward, transcription factor (TF) enrichment and the SCENIC analysis were performed on the Aβ-associated module to discover the related upstream regulators, and then a PPI network proximity method was employed to repurpose the potential approved drugs for AD.

**Results:**

A total of 16 co-expression modules were primarily obtained by the WGCNA method. Among them, the green module was significantly correlated with Aβ accumulation, and its function was mainly involved in neuroinflammation response and neuron death. Thus, the module was termed the amyloid-β induced neuroinflammation module (AIM). Moreover, the module was negatively correlated with neuron percentage and showed a close association with inflammatory microglia. Finally, based on the module, several important TFs were recognized as potential diagnostic biomarkers for AD, and then 20 possible drugs including ibrutinib and ponatinib were picked out for the disease.

**Conclusion:**

In this study, a specific gene module, termed AIM, was identified as a key sub-network of Aβ accumulation and neuroinflammation in AD. Moreover, the module was verified as having an association with neuron degeneration and inflammatory microglia transformation. Moreover, some promising TFs and potential repurposing drugs were presented for AD based on the module. The findings of the study shed new light on the mechanistic investigation of AD and might make benefits the treatment of the disease.

## 1. Introduction

Alzheimer's disease (AD) is a complex brain disorder that can explain nearly 60%−70% of worldwide dementia (Holtzman et al., 2011). With increasing prevalence and lacking effective treatment, more than 150 million people are estimated to be affected by AD in 2050 (GBD 2019 Dementia Forecasting Collaborators, 2022). Traditional drug research mostly focused on amyloid-β (Aβ), one extracellular hallmark of AD, but all failed except aducanumab that is approved by FDA in 2021, which is still controversial (Doig et al., 2017; Karran and De Strooper, 2022). On the other hand, neuroinflammation has been acknowledged as another important indication of events of AD development. A reduced AD risk is observed in clinics when anti-inflammatory drugs were used in some epidemiological research (Akiyama et al., 2000). As an inflammation response event in the brain of AD patients, Aβ accumulation was thought to be the key trigger of the disease. Some relevant targets and possible drugs have been presented but most of them do not gain the expected results in clinics due to the poor understanding of neuroinflammation caused by Aβ accumulation (Miguel-Álvarez et al., 2015; Fu et al., 2019; Dhapola et al., 2021). Therefore, it is necessary to systematically investigate the underlying network of neuroinflammation induced by Aβ which could facilitate to deeply comprehend the AD pathological mechanism and find a possible therapeutic approach.

**Figure 1:**
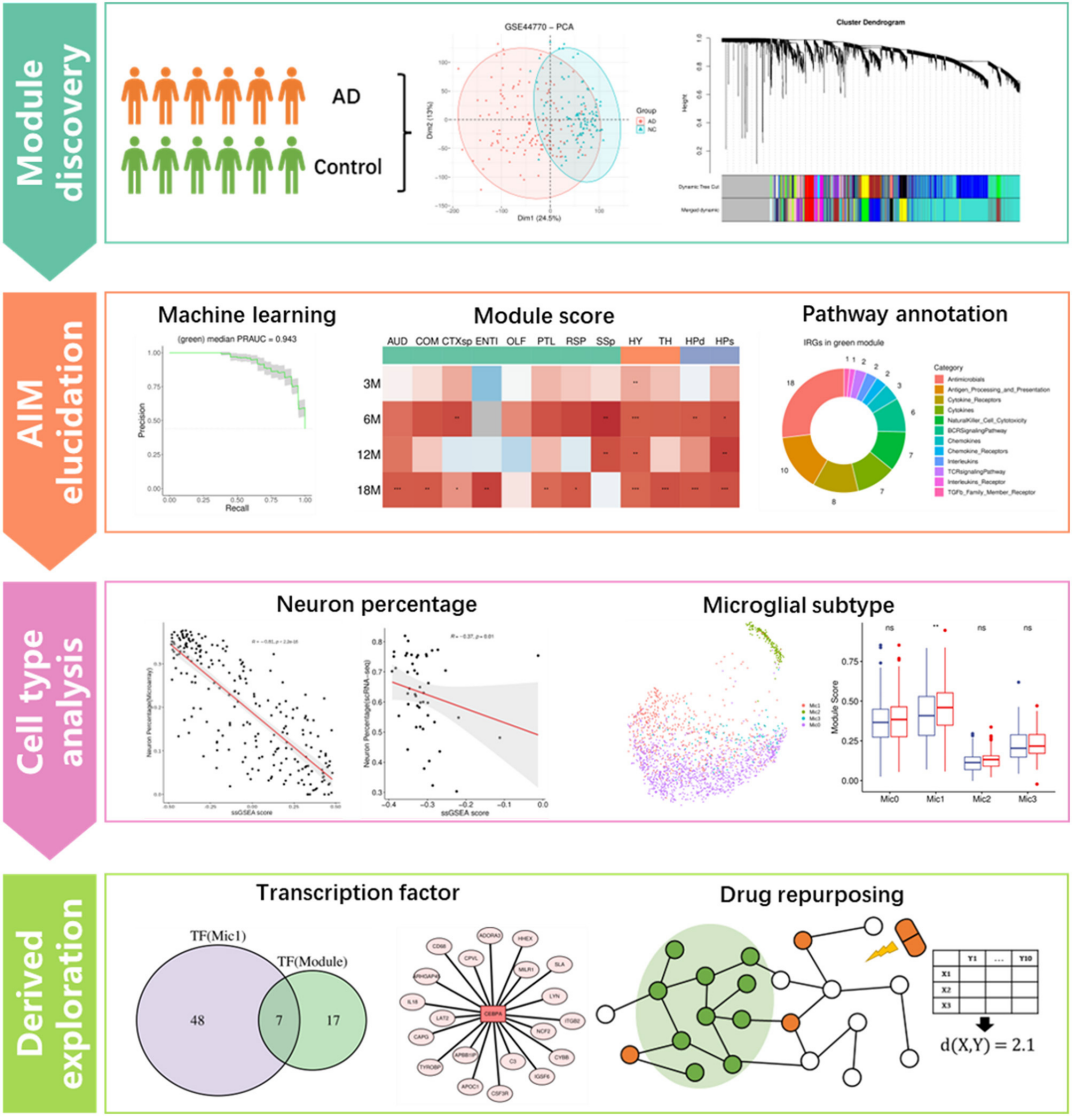
Flowchart of this study, which includes four main analysis steps. *p ≤ 0.05, **p ≤ 0.01, ***p ≤ 0.001.

Weighted gene co-expression network analysis (WGCNA) is an effective method to infer the trait-specific functional gene regulatory network (GRN) based on transcriptomic data (Langfelder and Horvath, 2008). For example, Feng et al. (2022) identified a cancer-associated fibroblast (CAF)-related module for ovarian cancer through the method. Lin et al. (2021) investigated the calcium signaling pathway-related GRN in ischemic stroke using the method. Additionally, the technology of single-cell RNA sequencing (scRNA-seq) has been developed in recent years to obtain the transcriptomic characteristics of individual cells in a tissue, which greatly contributes to discovering the key cell populations in a specific biological state (Kolodziejczyk et al., 2015; Andrews et al., 2021). For example, Obradovic et al. (2021) explored the cellular atlas of tumor microenvironment in clear cell renal carcinoma and identified the infiltrating macrophage subtype by comparing scRNA-seq data of the tumor tissue and the corresponding adjacent normal tissue. Derived analytic strategies such as cell communication analysis and master regulon inference will be of great benefit to understanding the roles of specific cell populations in a certain disease (Jin et al., 2021; Kumar et al., 2021). Compared to scRNA-seq, single-nucleus RNA-seq (snRNA-seq) is more suitable for frozen or hard-to-dissociate samples, especially brain tissues (Lake et al., 2016). In addition, spatial transcriptome (ST) provides precise transcriptomic heterogeneity of adjacent small spots in tissue (Longo et al., 2021). In this study, module identifying and drug repositioning analysis were conducted to elucidate the underlying mechanism of AD and find potential repurposing drugs for the disease. As the study flowchart showed (Figure 1), we first constructed the gene regulatory networks based on the transcriptomic data of AD patients and normal controls through the WGCNA method. Then, an Aβ-induced neuroinflammation module for AD was picked out based on the expression score and functional information. Afterward, the module was correlated with the neuron and distinct subtype of microglia to reveal its possible roles in the process of neuroinflammation development. Finally, several transcription factors regulating the module were presented as biomarkers for AD diagnosis, and 20 repurposing drugs against AD neuroinflammation were provided.

## 2. Materials and methods

### 2.1. Public transcriptomic data collection

AD-related transcriptomic data (detailed information is shown in Table 1) were mainly downloaded from the Gene Expression Omnibus (GEO, https://www.ncbi.nlm.nih.gov/geo) database. In practice, three microarrays (GSE44768, GSE44770, and GSE44771) datasets of the cerebellum (CR), dorsolateral prefrontal cortex (PFC), and visual cortex (VC) were collected, which included 129 late-onset Alzheimer's disease (LOAD) patients and 101 healthy controls. The probe ID was transformed into a gene symbol based on GPL4372 platform annotation information. Moreover, RNA-seq data with TPM normalized of the frontal cortex (BA9) from 120 normal brain samples (age >60) were downloaded from the Genotype-Tissue Expression project (GTEx, https://gtexportal.org/home/datasets). In addition, we obtained spatial transcriptomic data on 12 anatomical brain regions of App^NL − G−F^ KI and C57Bl/6J mice in 3, 6, 12, and 18 months (GSE152506), where each spot covered tissues with a diameter of 100 μm. Noteworthily, the Aβ plaque condition of these spots was inferred according to immunostaining assays of adjacent brain sections. Furthermore, we collected snRNA-seq data of the PFC region (BA10, containing 24 AD samples and 24 control samples) from the Synapse database (syn18485175).

**Table 1:** Public transcriptomic datasets used in this study.

**Type**	**GSE**	**GPL**	**Organism**	**Selected sample information**
Microarray	GSE44768	GPL4372	*Homo sapiens*	230 cerebellum (CR) samples from LOAD patients and healthy controls
Microarray	GSE44770	GPL4372	*Homo sapiens*	230 dorsolateral prefrontal cortex (PFC) samples from LOAD patients and healthy controls
Microarray	GSE44771	GPL4372	*Homo sapiens*	230 visual cortex (VC) samples from LOAD patients and healthy controls
RNA-seq	GTEx V8	/	*Homo sapiens*	212 normal brain frontal cortex (BA9) sample
snRNA-seq	Syn18485175	/	*Homo sapiens*	Prefrontal cortex (BA10) from 24 AD individuals and 24 non-AD individuals
Spatial transcriptomics	GSE152506	GPL19057	*Mus musculus*	2D-RNAseq on coronal section of App^NL − G−F^ KI mice and C57Bl/6J mice at 3,6,12, and 18 months of age

### 2.2. Weighted gene co-expression network analysis

Principal component analysis (PCA) was performed to check the difference between the AD and control group samples. Then, the weighted gene co-expression network analysis (WGCNA) was applied according to the recommended pipeline (https://horvath.genetics.ucla.edu/html/CoexpressionNetwork/Rpackages/WGCNA/Tutorials/index.html, WGCNA package of R software, version 1.71). In practice, genes with higher standard deviation among all samples were first filtered. Then, we removed distinct outlier samples by the “cutreeStatic” function, and the threshold of the scale-free topology index was set to 0.9. Afterward, the co-expression modules were identified by the “blockwiseModules” function, where the default parameters were used except for setting “corType” to “bicor” and “mergeCutHeight” to 0.1. Next, we calculated the correlation between each module and sample trait (AD or control) based on module eigengenes.

### 2.3. Module hub gene identification and logistic regression analysis

The unassigned gray module and modules with a large size (over 1,000 genes) were first dropped from the WGCNA result. Then, for the genes of the remaining modules, their module membership (known as kME) was evaluated by calculating the expression correlation with module eigengenes. The higher kME value (0–1) for a gene module pair means the gene is an important (central) element in the module. Therefore, the top 10 genes with the highest kME of each module were marked as hub genes.

For logical regression analysis, we used module hub gene expression as features and sample grouping as a binary indicator variable. AD patients were labeled “1” and healthy controls were labeled “0.” The R package mlr3 (version 0.13.4) was applied to build the logical regression model. The metric of Area Under the Precision-Recall Curve (AUPRC) was used for robust evaluation, and the median of three replicates of five-fold cross-validation (CV) was calculated to compare the performance of different models.

### 2.4. Calculation of module expression score

For microarray or RNA-seq datasets, the single-sample Gene Set Enrichment Analysis (ssGSEA) was performed to obtain the relative expression intensity of target modules. In practice, the “gsva” function (“ssgsea” method) of the GSVA package (version 1.42.0) was utilized to calculate the module score based on the expression matrix. For microglia cells in snRNA-seq data and spatial transcriptome datasets, expression count matrixes were first loaded into the Seurat package (version 4.1.1) and then normalized mainly for the library-size effect. Next, the “AddModuleScore” function was applied to calculate the expression score of modules for each cell or spot. As for each sample of the snRNA-seq data, their expression count matrixes were summarized to pseudo-bulk RNA-seq expression matrixes, and then the GSVA package was applied to calculate the overall expression score for the target module.

### 2.5. Functional annotation for genes of target modules

Seventeen immune-related gene (IRG) lists including 1,793 genes were first downloaded from the ImmPort website (https://www.immport.org/resources). Meanwhile, three classic pathway sets [including Gene Ontology Biological Process (GOBP), Kyoto Encyclopedia of Genes and Genomes (KEGG), and Reactome Pathway] were collected from the MsigDB database (https://www.gsea-msigdb.org/gsea/msigdb/). Then, the clusterProfiler package (version 4.2.2) was utilized to perform functional enrichment analysis based on pathway sets for genes of target modules, where the adjusted *p*-value threshold was set to 0.05. Additionally, human protein–protein interaction (PPI) information was extracted from the STRING database (https://string-db.org), where the combined score threshold was set to 600 to obtain credible interactions.

### 2.6. Cell-type enrichment analysis

The xCell package (version 1.1.0) was utilized to perform the cell-type enrichment analysis and predict neuron percentage for each sample of the microarray dataset. Then, the Spearman correlation coefficient between neuron percentage and the module score or expression of hub genes was calculated.

### 2.7. snRNA-seq data preprocessing and analysis

The snRNA-seq data were processed with the two-dimensional Uniform Manifold Approximation and Projection (UMAP) method implemented in the Seurat package (version 4.1.1) of R software to display cell types. Then, cell types were annotated based on the description of the original manuscript (Mathys et al., 2019), which included eight cell types, i.e., excitatory neurons (Ex), inhibitory neurons (In), microglia (Mic), astrocytes (Ast), oligodendrocytes (Oli), oligodendrocyte progenitors (Opc), endothelial (End), and pericytes (Per). Among them, microglia were further divided into four subtypes, namely, Mic0, Mic1, Mic2, and Mic3. Subsequently, for each sample of the snRNA-seq dataset, the correlation between neuron percentage and the module score was calculated. Then, the module score of cell types and microglia subtypes was compared between AD and control samples. Moreover, marker genes (average log_2_FC >0.5, adjusted *p*-value <0.05) and related biological pathways were identified for each microglia subtype. In practice, Fisher's exact test was performed to explore the link between microglia subtype and module genes. Additionally, pseudo-time trajectory among microglia subtypes was inferred using the monocle package (version 2.26.0). Finally, master regulons of microglia subtypes were inferred through the pySCENIC software (version 0.11.2). In detail, the network inference and motif enrichment were first performed to obtain potential regulons and target genes and then the expression activity of each regulon was evaluated through the Aucell function of the software.

### 2.8. Transcription factor enrichment analysis

A web-based tool, ChEA3 (https://maayanlab.cloud/chea3), was used to infer the relevant regulatory TFs for interested module genes, where the ReMap library was selected as the reference and Fisher's exact test was utilized as the statistical method.

### 2.9. Information collection for drugs and targets

Approved small molecular drugs were acquired from the TTD website (http://db.idrblab.net/ttd). Protein targets of these drugs were retrieved from the ChEMBL database (https://www.ebi.ac.uk/chembl). When a target's pChEMBL value was over 6, it was considered an effective one. Compound ID transformation between PubChem CID and ChEMBL ID was done through the PubChem Identifier Exchange Service (https://pubchem.ncbi.nlm.nih.gov/idexchange/idexchange.cgi).

### 2.10. PPI network proximity calculation from drug targets to hub genes of the interested module

A refined network proximity calculation method was adopted to evaluate the relevance between drugs and the interested module according to a previous study (Cheng et al., 2019). Here, **X** denoted all targets of a drug, **Y** denoted hub genes of the interested module, and **d**(**x, y**) represented the shortest PPI distance from a specific drug target (**x**) to one hub gene (**y**) of the interested module, while **D**(**X, Y**) reflected the average PPI distance for a drug from its targets to the hub genes of the module. The smaller the value of **D**(**X, Y**), the bigger the probability of a drug effect on the module. To evaluate the significance of the **D**(**X, Y**) value, background distributions were generated by randomly selecting the same number of pseudo-targets 1,000 times for a specific drug. These distributions were validated to conform to the Gaussian characteristics and ***μ*** and ***σ*** represent the mean and standard deviation, respectively. Subsequently, the **Z** score and corresponding *p*-value were calculated for **D**(**X, Y** ):


$$D\left(X,Y\right)\;=\;\frac{\sum_{x\in X}\sum_{y\in Y}d(x,y)}{\left\|X\right\|\times \left\|Y\right\|}\;,$$

$$\begin{array}{l} \text{Z}\;=\;\frac{\text{D}\left(\text{X},\text{Y}\right)-\mu }{\sigma }\;. \end{array}$$

## 3. Results

### 3.1. WGCNA identified 16 modules and related hub genes from the PFC region

**Figure 2:**
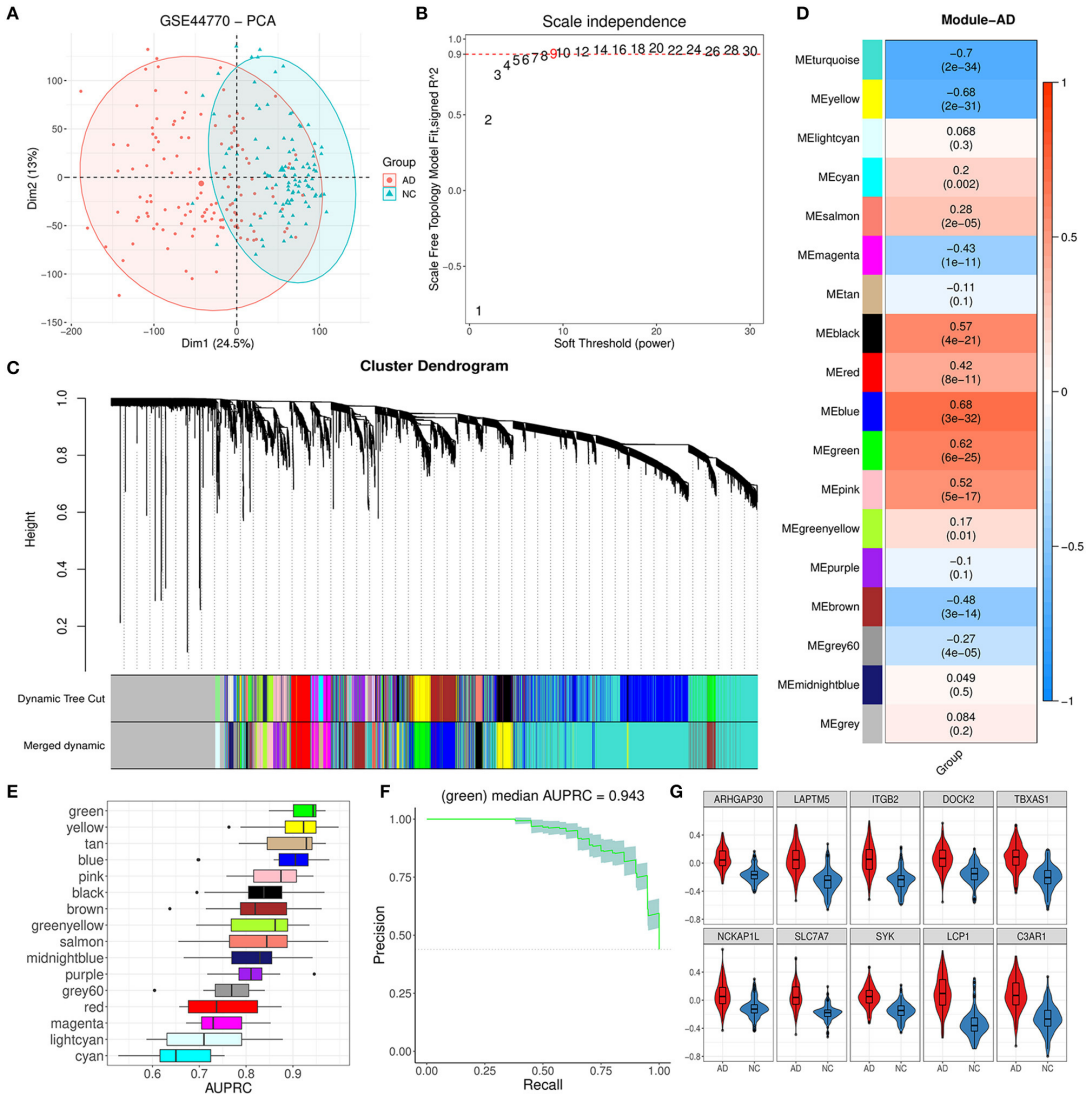
Module identification by WGCNA and primary exploration. **(A)** PCA dimension reduction plot for the difference between AD and NC samples in GSE44770. **(B)** Diagram displaying different soft-thresholding power and corresponding scale-free fit index. **(C)** Cluster dendrogram of genes with dissimilarity based on the topological overlap before and after merging. **(D)** Correlation heatmap between module eigengene and AD grouping. Each cell denoted the corresponding correlation and p-value for each module. **(E)** AUPRC distribution of cross-validation (CV) for 16 valid modules. **(F)** Precision–recall curve for the green module. The gray area represented the confidence interval of the CV. **(G)** Expression difference for 10 hub genes of the green module between AD and NC.

The PFC region expression data of 129 AD and 101 control brain samples from GSE44770 were used to construct the co-expression network. Based on probe annotation, an expression matrix comprising 230 samples and 19,870 genes was presented. The PCA result indicated that there was an obvious difference in the first principal component between groups (Figure 2A). Then, five outlier samples (over threshold 25 in hierarchical clustering) were dropped, and 5,000 highly variable genes were identified. We opted nine for the best soft-threshold leading to the eligible scale independence (Figure 2B). After the WGCNA analysis, the primary 18 modules were obtained (Figure 2C). Among these modules, the unassigned module (gray module) and module with too many genes (turquoise) were discarded, and thus, 16 modules were kept for further analysis. For these remainder modules, the top 10 hub genes for each module were identified subsequently according to strong eigengene-based connectivity (kME value; Supplementary Table 1).

### 3.2. Trait analysis found that the green module was abnormal in AD patients

As Figure 2D showed, the module eigengene of blue, green, pink, and black had a significant positive correlation to AD while yellow and brown modules exhibited opposite characteristics. To further reveal the most relevant module to AD, we evaluated the AD discrimination capability for each module based on expression information of their hub genes *via* the logistic regression model. According to the results of AUPRC assays, we found that the green module was the best one for AD discrimination among 16 modules with a performance median of 0.943 (Figures 2E, F). Afterward, we inspected the expression of the top 10 hub genes (ARHGAP30, LAPTM5, ITGB2, DOCK2, TBXAS1, NCKAP1L, SLC7A7, SYK, LCP1, and C3AP1) of the module and found that they were all upregulated significantly (Figure 2G), comparing the AD group with the control group. These results implied that the green module could play a vital role in AD progression.

### 3.3. Module scoring revealed that the green module was correlated with Aβ accumulation

**Figure 3:**
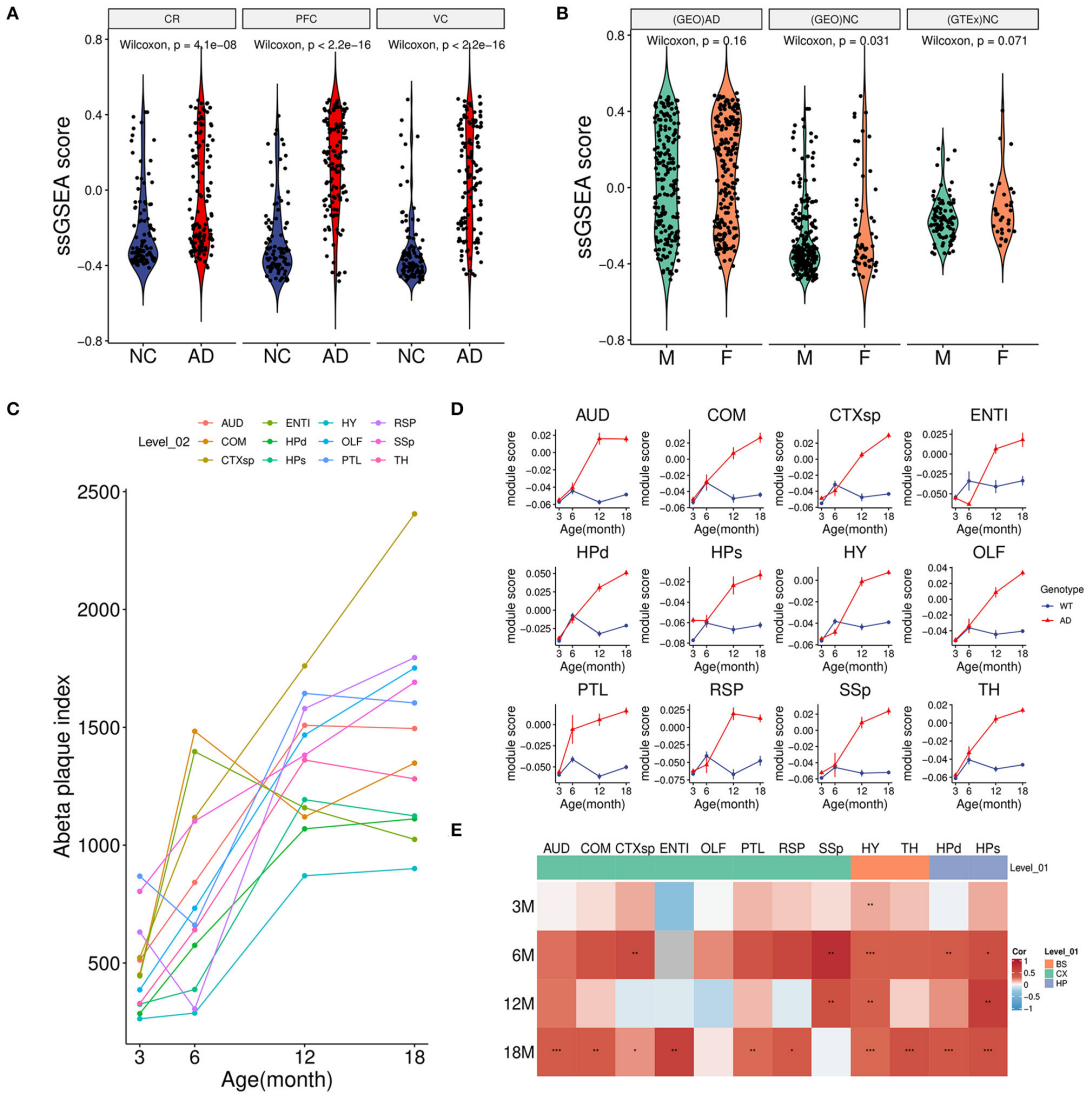
Association between module activity and Aβ. **(A)** Boxplot of the green module ssGSEA score difference between AD and NC among three brain regions. CR, cerebellum (GSE44768); PFC, dorsolateral prefrontal cortex (GSE44770); VC, visual cortex (GSE44771). **(B)** Boxplot of the green module ssGSEA score difference between male (M) and female (F) in different groups. (GEO)AD, all AD patients of above microarray data from GEO; (GEO)NC, all healthy controls of above microarray data from GEO; (GTEx)NC, normal brain samples from the GTEx project. **(C)** Line plot showed an average Aβ plaque index of different age stages in AD model mice among 12 brain regions. For detailed region information, refer the link (https://alzmap.org). **(D)** Line plots of the green module score difference at different age stages between the AD model and control mice among 12 brain regions. **(E)** Correlation heatmap of the Aβ plaque loading index and green module score between different age stages and brain regions in AD model mice. *p < 0.05, **p < 0.01, ***p < 0.001.

To further investigate the relationship between the green module with AD, we calculated the overall activity of the module and found that the ssGSEA score in AD was considerably higher than that of the control group not only in the PFC region but also in CR(GSE44768) and VC(GSE44771) regions (Figure 3A). These findings implied that the green module was activated sharply in multiple brain regions under AD conditions. Moreover, there was no obvious difference in the gender of the disease for the module (Figure 3B). Normal brain data from GTEx also confirmed that the green module was independent of gender (Figure 3B).

Combined with mice spatial transcriptome data (GSE152506), the correlation between the module and amyloid plaque was further explored. First of all, increasing amyloid deposition was observed in most brain regions of the AD model mice from 3 to 18 months (Figure 3C). Correspondingly, we found that the expression of the green module in the AD group showed a significantly increasing trend during the period (Figure 3D). However, these regions in the control group maintained relatively low expression activity. It was worth noting that the module score of AD and control groups was very similar in the early stage which implied that long-term and increasing-intensity stimulation from Aβ could be the main reason for the high expression of the module genes across many brain regions. Moreover, in the old model (18-month-old) mice of the AD group, a strongly positive correlation of module activity with the plaque index in most regions further verified that amyloid accumulation might be responsible for gene expression of the green module (Figure 3E).

### 3.4. Functional annotation showed that the green module was involved in neuroinflammation

**Figure 4:**
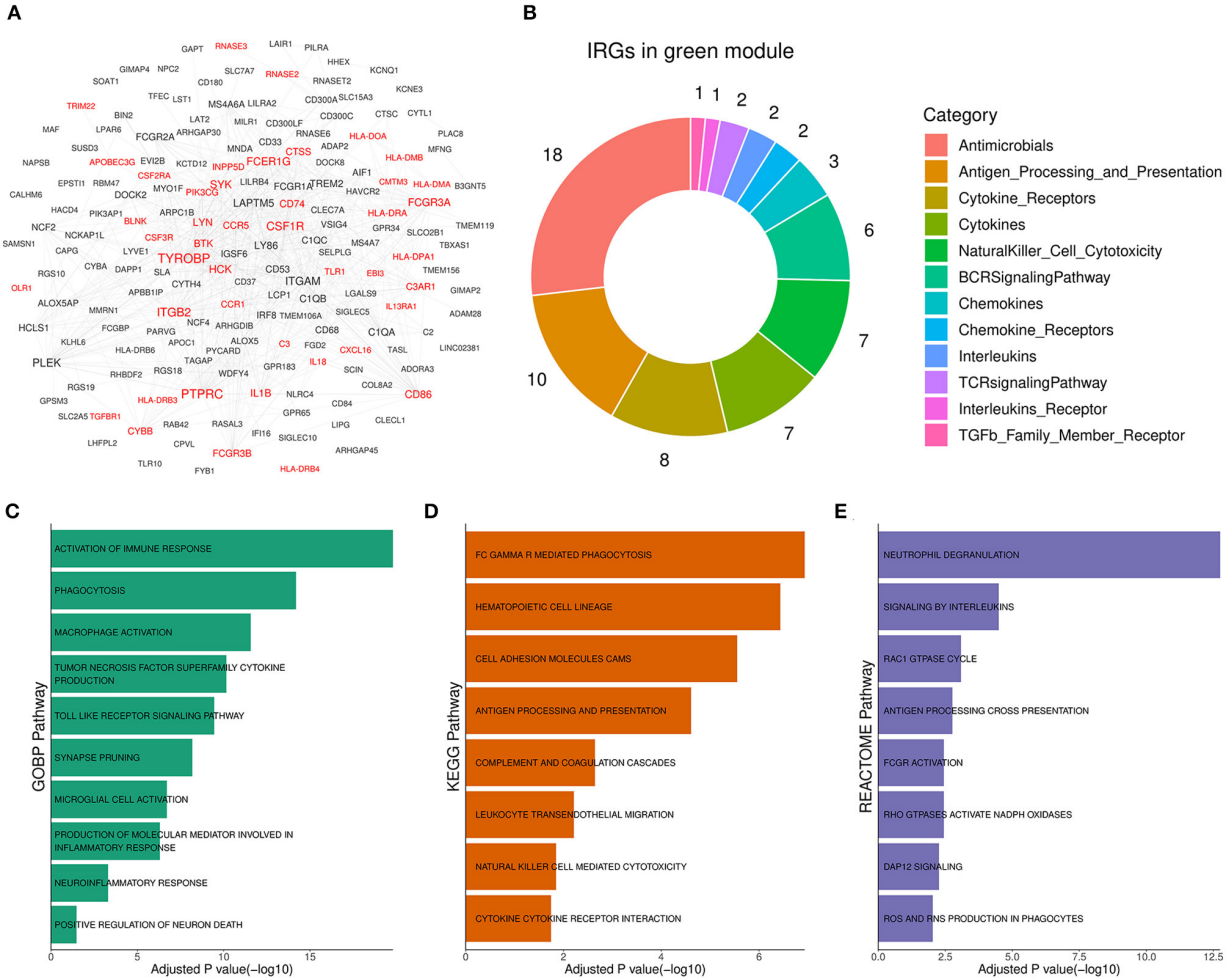
Functional annotation for the green module. **(A)** Diagram for the PPI network of the green module. Labels with red color represented immune-related genes (IRGs). A combined score above 600 was considered as a reliable PPI link. **(B)** Donut chart of the immune categories that IRGs in the green module involved. Enrichment analysis for green module genes using **(C)** GOBP, **(D)** KEGG, and **(E)** Reactome pathway set, respectively. The threshold of BH adjusted *p*-value was set to 0.05.

To reveal the role of the green module in AD progression, functional annotation was conducted for the genes of the module. According to annotation results, 44 out of the 177 genes in the green module (Figure 4A, Supplementary Table 2) were immune-related genes, and they were involved in antimicrobe, antigen processing and presentation, cytokine signal transduction, and natural killer cell cytotoxicity (Figure 4B). Subsequently, we performed pathway enrichment analysis based on different pathway sets (Supplementary Table 3). Biological process analysis results showed that genes of the module were mainly associated with TNF superfamily cytokine production, toll-like receptor signaling, synapse pruning, neuroinflammation, and neuron death response (Figure 4C). The signal pathway detection result was focused on FC GAMMA-mediated phagocytosis, cell adhesion molecules CAMs, complement, and coagulation cascades (Figure 4D). Moreover, the significant pathway from Reactome showed that the module was related to interleukin signaling, DAP12 signaling, and ROS and RNS production (Figure 4E). Above enriched pathways implied that the gene overexpressed abnormally in the green module could activate unexpected immune responses in the brain and boost neuroinflammation, which was harmful to normal neurons and synapses. Therefore, the green module was termed the Aβ-induced neuroinflammation module (AIM) based on the above analysis results.

### 3.5. Correlation analysis showed neuron and microglia was associated with the AIM

**Figure 5:**
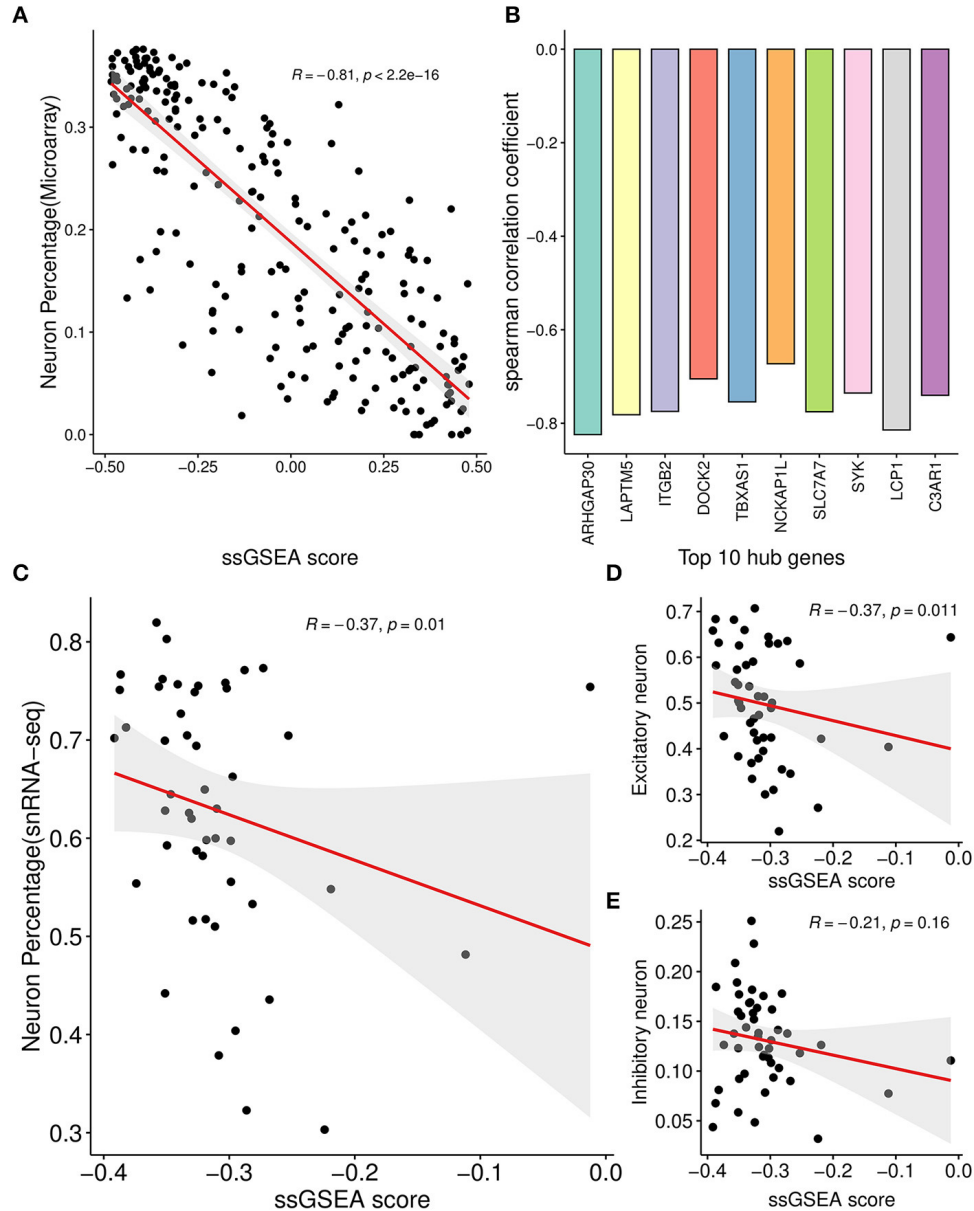
Correlation analysis between AIM and neuron percentage. **(A)** Scatter plot of the relationship between the module ssGSEA score and neuron percentage predicted by the xCell algorithm in GSE44770 microarray samples. *R* represented the Spearman correlation coefficient. **(B)** Bar plot showing the Spearman correlation coefficients between 10 module hub genes and neuron percentage predicted by the xCell algorithm in GSE44770 microarray data. **(C)** Scatter plot of the relationship between the module ssGSEA score and neuron percentage in syn18485175 snRNA-seq samples. **(D, E)** were similar and based on excitatory and inhibitory neurons, respectively.

**Figure 6:**
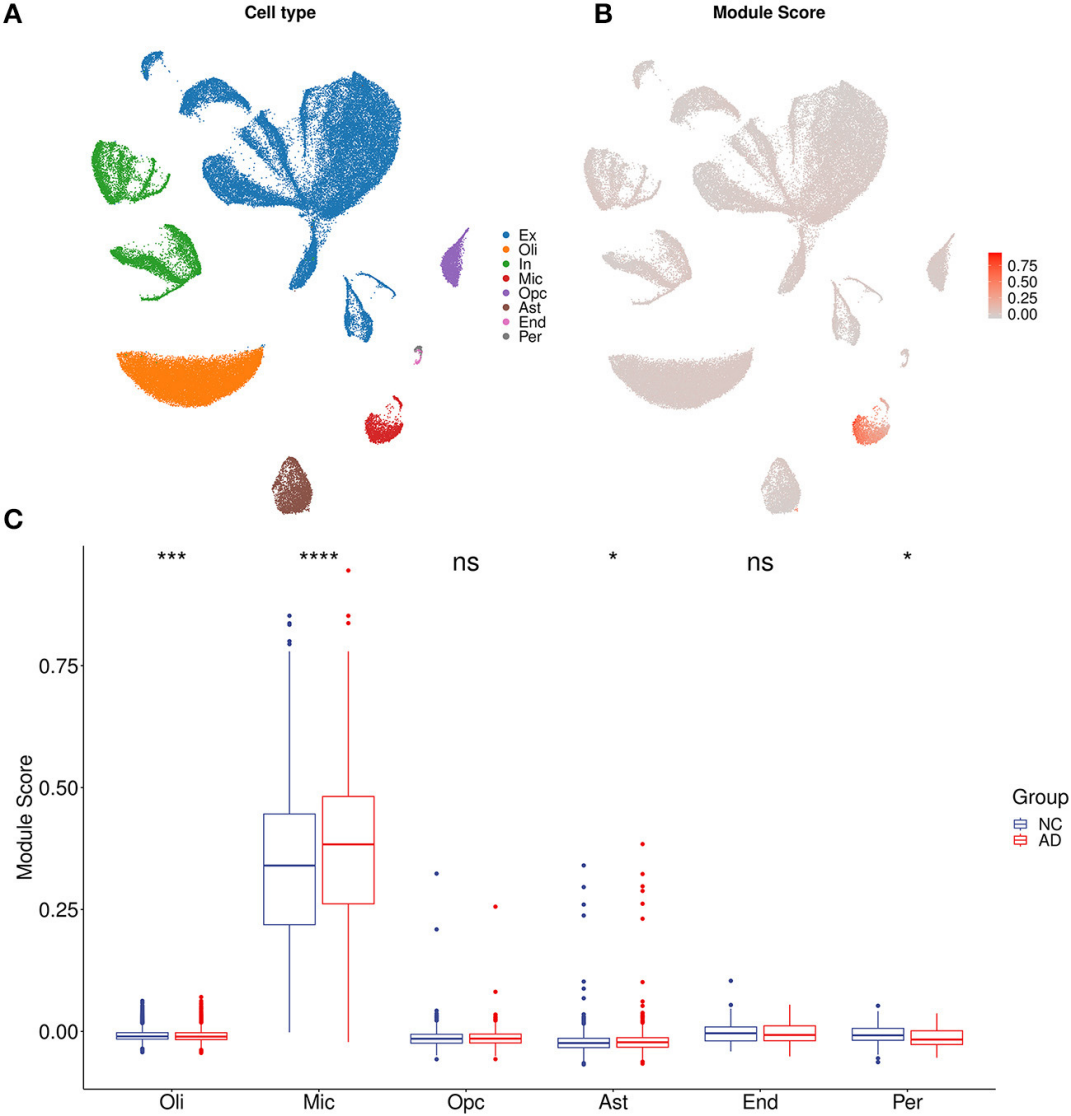
AIM-related cell type analysis. **(A)** UMAP plot of eight primary cell types in syn18485175 snRNA-seq. Ex, excitatory neurons; In, inhibitory neurons; Mic, microglia; Oli, oligodendrocytes; Opc, oligodendrocyte progenitors; Ast, astrocytes; End, endothelial; Per, pericytes. **(B)** UMAP plot of snRNA-seq. Each cell was colored according to AIM expression. **(C)** Bar plot showing AIM expression in different cell types. Blue denotes the control group and red denotes the AD group. The Wilcoxon test method was used to compare the group difference. ns: *p* > 0.05, *p ≤ 0.05, ***p ≤ 0.001, ****p ≤ 0.0001.

For each sample of the microarray dataset, neuron percentage was first predicted, and then its correlation with the AIM score was calculated. Calculation results showed that there existed a significantly negative correlation between neuron percentage and AIM score (*R* = −0.81, *p* < 0.05, Figure 5A). Furthermore, correlation coefficients of the top 10 hub genes of AIM were calculated, respectively, and they were all below −0.6 (Figure 5B). While for samples of the snRNA-seq dataset, a negative correlation with the AIM score was also observed in the overall neuron (Figure 5C), excitatory neuron (Figure 5D), and inhibitory neuron (Figure 5E). These results validated that AIM upregulation could be responsible for neuron degeneration in AD. In order to understand the potential mechanism, we investigated the association between AIM and other cell types. A remarkable AIM score was exclusively observed in microglia (Figures 6A, B). A significantly increased AIM score was identified for the AD group as compared to the control group in oligodendrocytes, microglia, and astrocytes (Figure 6C). These results suggested that AIM was associated with neuron reduction and microglia transition.

**Figure 7:**
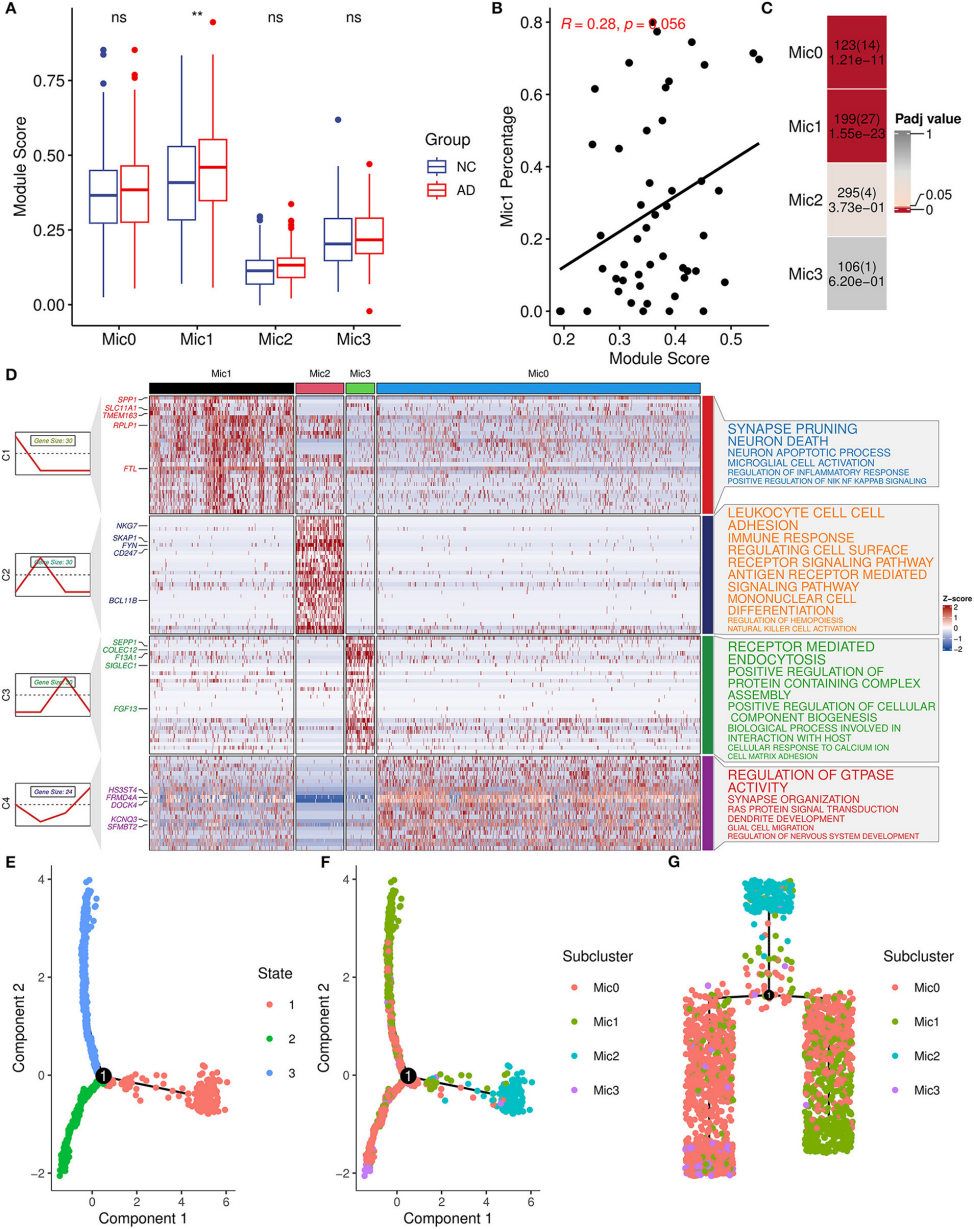
AIM-related microglia subcluster analysis. **(A)** Bar plot showing AIM expression in different microglia subclusters. Blue denotes the control group and red denotes the AD group. The Wilcoxon test method was used to compare the group difference. ns: *p* > 0.05, **p ≤ 0.01. **(B)** Scatter plot of the relationship between the module score and Mic1 percentage in the snRNA-seq microglia population. *R* represents the Spearman correlation coefficient. **(C)** Heatmap of Fisher's exact test for the overlapped genes from the AIM to different microglia subtype marker genes. The numbers in each grid denote marker gene size, overlapped gene size, and adjusted *p*-value. **(D)** Heatmap of scaled gene expression in different microglia subtypes. C1, C2, C3, and C4 represent marker genes of Mic1, Mic2, Mic3, and Mic0, respectively. The top five marker genes for each subtype were labeled. The right panel represented the enriched GO biological pathways of the corresponding marker gene set. The larger text size represented more significant enrichment results. **(E)** Trajectory plot of cells in the reduced dimensional space. Each cell was colored by inferred states and **(F)** was colored by microglia subclusters. **(G)** Trajectory tree of cells and each cell was colored by microglia subclusters.

Considering the heterogeneity of microglia, microglia cells were divided into four subtypes (Mic0, Mic1, Mic2, and Mic3) and then the association between subtypes and AIM was explored. Mic0 and Mic1 subtypes showed a higher association score than other subtypes. In addition, Mic1 was the only subtype whose association score in the AD group was significantly greater than that in the control group (Figure 7A). Interestingly, for the sample of Mic1, the percentage of the microglia population was also positively correlated with AIM (*R* = 0.28, *p* = 0.056, Figure 7B). Subsequently, marker genes of subtypes were detected, and there were 123, 199, 295, and 106 marker genes in Mic0, Mic1, Mic2, and Mic3, respectively (Supplementary Table 4). Through Fisher's exact test, we found that the marker genes of Mic1 and Mic0 considerably overlapped with AIM genes (Figure 7C). Pathway enrichment analysis showed that marker genes of Mic1 were focused on synapse pruning, neuron death, neuron apoptotic process, regulation of inflammatory response, and positive regulation of NIK NF KAPPB signaling. While marker genes of Mic0 were involved in GTPASE activity regulation, synapse organization, RAS protein signal transduction, and glial cell migration (Figure 7D). Pseudo-time trajectory analysis revealed that Mic1 and partial Mic0 might be advanced subtypes of microglia cells (Figures 7E–G). These results implicated that Mic1 was a distinct subtype associated with AIM, which might provide new clues to neuroinflammation and neuron reduction in AD.

### 3.6. Transcription factor analysis found the vital upstream regulator of the AIM

**Figure 8:**
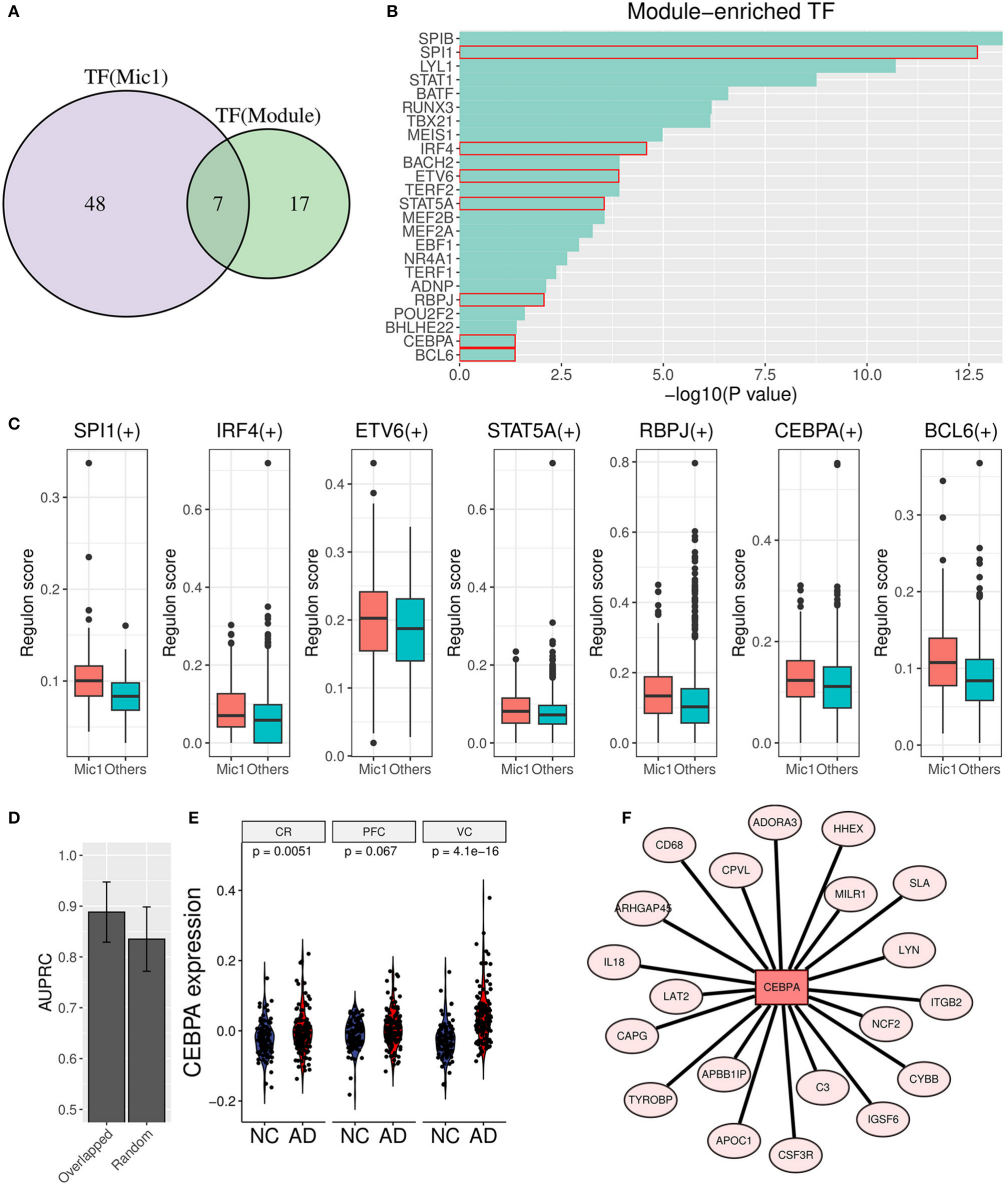
Transcription factor analysis for AIM. **(A)** Venn diagram displays the overlap between AIM-enriched TFs and Mic1-related TFs. **(B)** Bar plot of AIM-enriched TFs. The seven shared TFs were marked by a red box. **(C)** Boxplot of seven overlapped TFs involved regulon activity between the Mic1 subtype and other subtypes. **(D)** Bar plot of AUPRC distribution of above TFs or random seven human TFs based on GSE44770 microarray data. The model was based on a logistic algorithm. Selected TF expression was used as a feature, and sample grouping (AD or non-AD) was used as diagnostic targets. **(E)** Violin plot of CEBPA expression difference between NC and AD is based on three microarray datasets (GSE44768, GSE44770, and GSE44771). **(F)** TF network diagram shows CEBPA and its target genes in the AIM.

To unearth the underlying upstream transcription factors (TFs) of AIM genes, TF enrichment analysis was performed based on ReMap library (Keenan et al., 2019), and 24 significant TFs (*p* value < 0.05) were obtained as a result. Simultaneously, Mic1-associated TFs were detected through the SCENIC analysis, and 55 TF regulons were picked out. Furthermore, seven overlapped TFs (SPI1, IRF4, ETV6, STAT5A, RBPJ, CEBPA, and BCL6) were identified as important upstream regulators (Figures 8A–C). Then, the diagnostic value in AD discrimination of these seven TFs was verified by comparing them with seven random human transcription factors (Figure 8D). Among them, CEBPA expression in the AD group was higher than that in the control group in three brain regions (Figure 8E). Its target genes in AIM included some known pro-inflammatory factors such as C3, IL18, CD68, and ITGB2 (Figure 8F). According to these findings, we speculated that CEBPA could be a crucial TF for AD involved in microglia-mediated neuroinflammation.

### 3.7. AIM-based repurposing method aided in anti-neuroinflammation drug discovery

**Figure 9:**
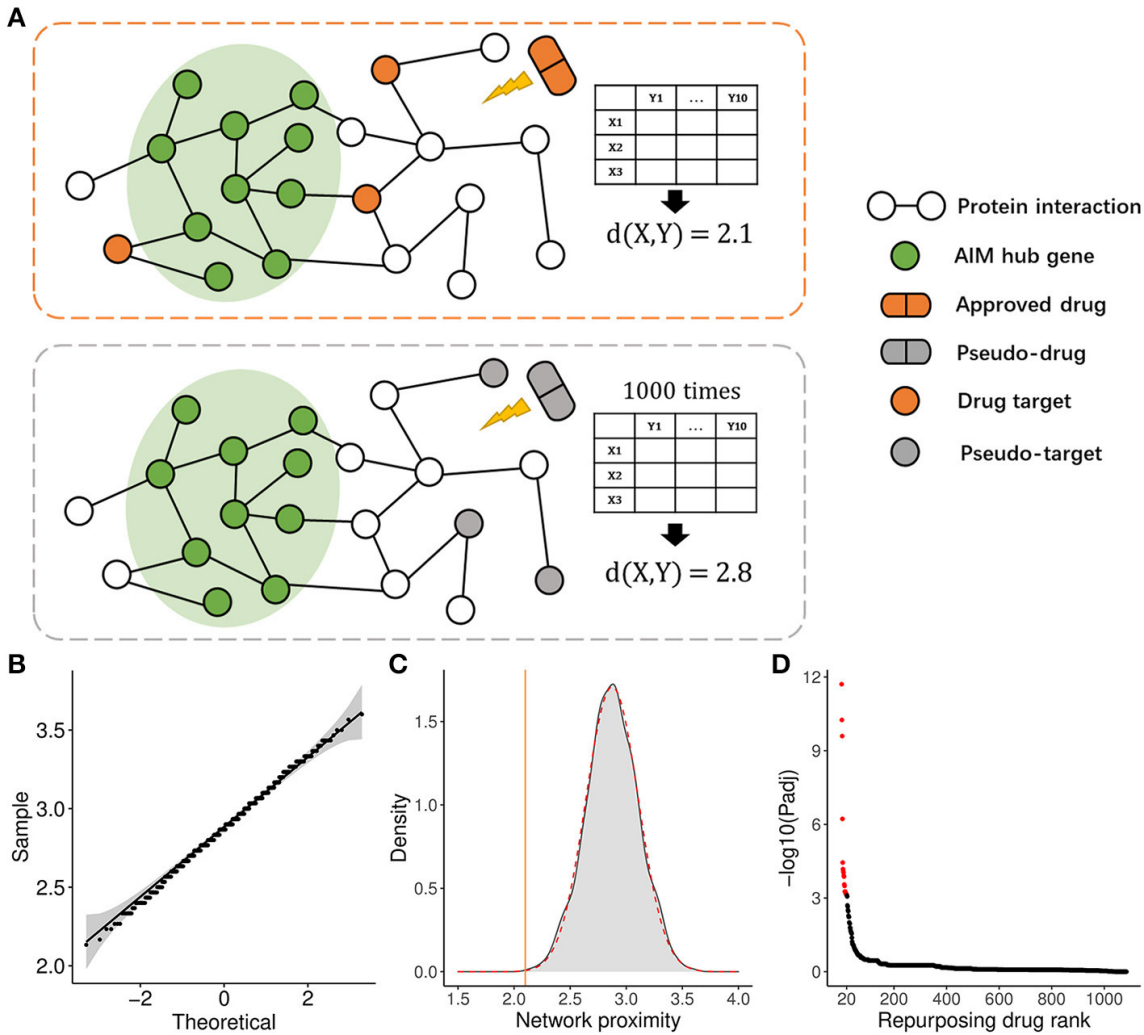
Drug repurposing method based on the AIM. **(A)** Diagram demonstrated the average PPI network proximity calculation method from all drug targets to module hub genes (e.g., *X* = 3, *Y* = 10). **(B)** Quantile-Quantile plot (QQ plot) indicated background distribution based on random pseudo-targets conformed to Gaussian distribution well. **(C)** Gray density plot represents background distribution based on random three pseudo-targets and the red dashed curve represents fitted Gaussian distribution. The brown line indicated the actual proximity of the drug. **(D)** The scatter plot showed the rank of 1,084 approved drugs based on the adjusted *p*-value.

To apply the therapeutic role of the AIM, we adopted a PPI network proximity method to estimate the possible effect of approved drugs for anti-neuroinflammation based on the AIM, which might subsequently benefit the treatment of AD. First, 1,084 approved small molecular drugs, and their literature-based protein targets were retrieved from public databases. Then, the average PPI distance from drug targets to the top 10 hub genes of AIM was calculated (Figure 9A). As for a specific drug, the possibility of anti-neuroinflammation was estimated by the *Z*-score and a *p*-value based on the corresponding background Gaussian distribution (Figures 9B, C). Ultimately, the top 20 potential drugs were presented according to the Benjamini–Hochberg (BH) adjusted *p*-value (Figure 9D, Table 2). Among these drugs, six drugs with an anti-neuroinflammation effect have been reported, which confirmed the validity of our method, while the other 14 drugs could be new potential small molecules for AD treatment.

**Table 2:** Top 20 repurposing drugs according to significantly close PPI network proximity.

**Rank**	**Drug ID**	**Drug name**	**Background distribution (Targets|Mean|SD)**	**Proximity**	**Padj**	**Literature**
1	D0H0EQ	Ponatinib	53|2.867|0.056	2.428	1.95E-12	No
2	D09KTS	Ibrutinib	30|2.868|0.073	2.330	5.59E-11	Yes
3	D0O0LS	Entrectinib	47|2.868|0.059	2.453	2.53E-10	No
4	D0E6XR	Dasatinib	95|2.867|0.041	2.627	5.97E-07	Yes
5	D0G6QF	Vandetanib	62|2.867|0.051	2.605	3.60E-05	No
6	D0J5VR	Idelalisib	7|2.875|0.156	2.100	6.64E-05	No
7	D0Q9EV	Lifitegrast	3|2.874|0.233	1.733	7.90E-05	No
8	D03ZBT	Crizotinib	106|2.867|0.039	2.678	9.25E-05	No
9	D0IQ6P	Cabozantinib	22|2.867|0.086	2.459	1.21E-04	No
10	D0S5LD	BAY 80-6946	6|2.875|0.172	2.067	1.33E-04	No
11	D04LVK	Ceritinib	23|2.868|0.084	2.487	2.80E-04	No
12	D09GDD	Regorafenib	24|2.869|0.082	2.500	3.21E-04	Yes
13	D09HNV	Intedanib	163|2.867|0.03	2.736	5.42E-04	No
14	D0RU0O	IPI-145	5|2.873|0.188	2.060	5.42E-04	No
15	D0W7HE	Alpelisib	5|2.873|0.188	2.060	5.42E-04	No
16	D0K8KX	Quercetin	34|2.868|0.068	2.579	6.51E-04	Yes
17	D0V9WF	Lestaurtinib	276|2.867|0.023	2.770	6.51E-04	No
18	D0W5HK	Sorafenib	74|2.867|0.047	2.666	6.51E-04	Yes
19	D01PZD	Romiplostim	11|2.873|0.125	2.345	7.25E-04	No
20	D0OB0F	Bosutinib	122|2.867|0.036	2.717	7.30E-04	Yes

## 4. Discussion

Alzheimer's disease is one of the most common neurodegenerative diseases, which is characterized by amyloid-β (Aβ) accumulation and phosphorylated tau (ptau) (Braak and Braak, 1991; Yuksel and Tacal, 2019). Aβ cascade hypothesis supposes amyloid plaque is a major cause of neuron death and synapse dysfunction (Hardy and Higgins, 1992). Although some drugs directly target Aβ that could reduce plaque loading, AD symptoms do not get the expected relief in clinical trials (Lannfelt et al., 2014). On the other hand, it is increasingly recognized that neuroinflammation is a vital event in AD onset and progression (Hammond et al., 2019). GWAS research studies show that some important AD risk genes are closely related to immune function and immune cells (Sims et al., 2017; Jansen et al., 2019). Simultaneously, emerging evidence proposes that amyloid plaque is responsible for microglia activation which can cause neuroinflammation (Cheng et al., 2021; Leng and Edison, 2021). However, due to complex immune signaling and microglia heterogeneity in the brain, immune-related therapies against AD neuroinflammation are limited at present. More systemic research is needed to explore the potential gene network of Aβ-induced neuroinflammation in AD, which might help to uncover novel biomarkers and provide a new treatment for the disease.

In this study, we successfully identified an Aβ-induced neuroinflammation module (AIM) for AD via the WGCNA method combined with module scoring and pathway enrichment analysis. In practice, microarray and snRNA-seq datasets were mainly focused on the PFC region, which was closely related to AD symptoms (Sampath et al., 2017; Sun et al., 2022). Primary WGCNA results based on the PFC microarray dataset indicated that the module comprising 177 genes was one of the modules most relevant to AD. Moreover, we also found amyloid plaque was the major factor that affected gene expression of the AIM in the spatial transcriptome of the AD model of mice. Genes' function of the module was mainly involved in the neuroinflammation-related process discovered by pathway enrichment analysis. Aβ, comprised of short peptides cleaved from amyloid precursor protein (APP), is an extracellular hallmark of AD. As its well-known toxicity of endogenous stimuli to neurons, an innate immune system like microglia in the brain is commonly activated to exert a protective response for Aβ accumulation. However, a long-term immune stimulus would cause a detrimental effect on the neuron and synapse due to the unexpected inflammation response in the brain.

As for genes in AIM, some are pro-inflammatory mediators including cytokines (IL1B and IL18) and chemokines (CXCL16, RNASE2, and CCR1), which can lead to neuronal dysfunction and death (Hanisch, 2002; Micheau and Tschopp, 2003). For example, IL1B is correlated to the loss of synaptic connections in rat hippocampus (Mishra et al., 2012). Some genes (C3, C1QA) about complement cascade play an important role in synapse refinement during brain development (Schafer et al., 2012), but aberrant upregulation and deposition of complement will lead to synapse loss and cognitive impairment. Some AD risk genes are also found in AIM, such as HLA-DRA, TREM2, MS4A6A, CD33, and PIK3CG. Recent studies have revealed that CD33 and TREM2 are the two most potential targets, which play a respective pro-/anti-neuroinflammation role during the AD process. Relevant immunotherapies of CD33 inhibiting and TREM2 elevating have made a little progress in a clinical trial (Griciuc et al., 2019; Griciuc and Tanzi, 2021). Additionally, other genes of AIM might be associated with AD neuroinflammation. For example, three hub genes (ARHGAP30, DOCK2, and NCKAP1L), NCF, and TAGAP are involved in the RAC1 GTPase cycle, which can regulate the production of neurotoxic reactive oxygen species (ROS) (Etienne-Manneville and Hall, 2002). To sum up, the AIM can be used to pinpoint key genes about Aβ-induced AD neuroinflammation and discover possible novel targets for the disease.

Afterward, we investigated AIM-related cell types to further explore their roles in AD. First of all, we found a negative correlation between AIM activity and neuron ratio in both microarray data and snRNA-seq data, which confirmed the potential link of the module to AD symptoms. Furthermore, we found that microglia showed a remarkable AIM score. Microglia is one resident innate immune cell type originating from a yolk sac progenitor and mainly exerts immune surveillance and clearance in the central nervous system (CNS) (Ousman and Kubes, 2012; Gomez Perdiguero et al., 2015). Its activation against endogenous or exogenous stimulation (such as abnormal aggregating of some essential proteins) is an important defensive response to reduce the currency of neurological disease (Heneka et al., 2015). However, microglia often show spatial and morphological heterogeneity which present complex roles and phenotypes in neurological diseases (Plescher et al., 2018; Tan et al., 2020). Moreover, its adverse activation is recognized as a risk event of neuroinflammation in AD (Yu and Ye, 2015; Nguyen et al., 2017). The original manuscript (Mathys et al., 2019) of snRNA-seq data identified four microglia subtypes, and Mic1 was a distinct microglia subtype that presented partial features of DAM, which was derived from the mouse model and reflected phagocytosis phenotype against Aβ particles (Keren-Shaul et al., 2017). In this study, we validated that AIM was considerably associated with Mic1, and then functional exploration revealed that the subtype could mediate neuroinflammation and induce neuron degeneration.

Next, through transcription factor (TF) enrichment analysis and single-cell regulatory network inference and clustering analysis, seven potential TFs related to AD (SPI1, IRF4, ETV6, STAT5A, RBPJ, CEBPA, and BCL6) were identified. Among these TFs, CEBPA (CCAAT/Enhancer-Binding Protein Alpha), containing a basic leucine zipper (bZIP) domain, can recognize the CCAAT motif in the promoters of interested target genes. It was found that CEBPA had a higher expression in AD samples compared to normal ones in three brain regions. It is confirmed that CEBPA plays an important role in the proliferation and differentiation of a myeloid progenitor, and its biallelic mutation is highly related to acute myeloid leukemia (AML) (Leroy et al., 2005; Wilhelmson and Porse, 2020). Emerging evidence has uncovered its role in microglia-associated neuroinflammation. For example, downregulated CEBPA is associated with anti-inflammation microglia (M2) polarization (Yu et al., 2017). Another research demonstrates that CEBPA can coordinate with other two TFs (IRF1 and LXR) to regulate pro-inflammation cytokine production in microglia stimulated by lipopolysaccharide (LPS) (Gao et al., 2019). Notably, they also found that siRNA against CEBPA can significantly inhibit the production of IL6, IL1b, and IL5. In addition, its targets gene in AIM includes inflammatory markers such as IL18, CD68, TYPOBP, and AD risk genes such as ARHGAP45 and APOC1 (Xue et al., 2021; Kulminski et al., 2022). In summary, these seven candidate TFs, especially CEBPA, could be used as potential biomarkers for AD diagnosis.

Finally, 20 potential drugs of anti-neuroinflammation against AD were presented based on significant network proximity. Some of them have been proven to have an anti-neuroinflammation effect, which reflected the validity of our method. For instance, ponatinib is a multi-target tyrosine kinase inhibitor and ranked first among all drugs. Previous studies reported that ponatinib can reduce inflammation of obesity and influenza (Chen et al., 2019; Lin et al., 2022) due to its inhibitory effect on the two isoforms of JAK (JAK1 and JAK2). The JAK/STAT pathway plays important roles in glial activation and neuroinflammation response in many neurodegenerative diseases (Jain et al., 2021). Additionally, ibrutinib is originally known as a bruton tyrosine kinase inhibitor (BTKi) and has bioactivity against other kinases (Cheng et al., 2014). It has been approved by FDA for multiple diseases such as mantle cell lymphoma (MCL) and chronic lymphocytic leukemia (CLL). A total of three genes (HCK, BTK, LYN) out of 30 Ibrutinib targets were found in AIM, and its proximity score ranked second among all drugs. Recent studies show that ibrutinib can attenuate neuroinflammatory responses by inhibiting AKT/STAT3 signaling pathways in the LPS-stimulated BV2 cell line and reduce glia activation and cytokine levels in animal experiments (Nam et al., 2018; Li et al., 2021). Another research further indicates that the anti-neuroinflammation effect of ibrutinib is associated with Aβ accumulation in 5xFAD mouse models (Lee et al., 2021). In addition, regorafenib, ranked 11th, has been proven as having effects on neuroinflammation suppression and dendritic spine formation (Han et al., 2020).

There were also some limitations in our study. For 177 constituent genes in the AIM, it is important to exclude the possible false positive genes and further recognize minor submodules that could participate in Aβ-induced neuroinflammation in different roles during the AD process. Moreover, a prospective study for identified novel biomarkers and screened candidate drugs is worthy of deep investigation through molecular biology experiments.

## 5. Conclusion

We identified a vital Aβ-induced neuroinflammation module (AIM) made up of 177 genes for AD. The neuron reduction and AIM-related inflammatory microglia subtype were further discovered to elucidate the roles of the module. Moreover, some potential TF biomarkers and some candidate repurposing drugs were presented. In short, our findings provided sights into the gene regulatory network and drug targets of AD neuroinflammation, which might facilitate mechanistic investigation of AD and make benefits to treatment of the disease.

## Data availability statement

The public sequencing data presented in the study are deposited in the GEO repository: https://www.ncbi.nlm.nih.gov/geo/. GSE44768: https://www.ncbi.nlm.nih.gov/geo/query/acc.cgi?acc=GSE44768; GSE44770: https://www.ncbi.nlm.nih.gov/geo/query/acc.cgi?acc=GSE44770; GSE44771: https://www.ncbi.nlm.nih.gov/geo/query/acc.cgi?acc=GSE44771; GSE160936: https://www.ncbi.nlm.nih.gov/geo/query/acc.cgi?acc=GSE160936; GSE152506: https://www.ncbi.nlm.nih.gov/geo/query/acc.cgi?acc=GSE152506 and Synapse repository: https://www.synapse.org/Syn18485175: https://www.synapse.org/#!Synapse:syn18485175.

## Author contributions

SL, DL, and GZ designed the study and wrote the manuscript. SL, CL, and ZZ collected and analyzed the data. All authors contributed to the article and approved the submitted version.
